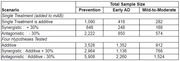# The impact of lecanemab and donanemab on future Alzheimer’s disease clinical development

**DOI:** 10.1002/alz.093476

**Published:** 2025-01-09

**Authors:** Samuel P. Dickson, Craig Mallinckrodt, Aaron H Burstein, Laura Nisenbaum, Howard M Fillit, Suzanne B. Hendrix

**Affiliations:** ^1^ Pentara Corporation, Salt Lake City, UT USA; ^2^ Alzheimer’s Drug Discovery Foundation, New York, NY USA

## Abstract

**Background:**

A new era of Alzheimer’s disease (AD) research is beginning now that multiple monoclonal antibodies (mABs) are on the market. Their use may not be widespread initially but will be more common in clinical sites likely to participate in clinical trials and will continue to grow. Many AD investigational treatments have been studied as add‐on to acetylcholinesterase inhibitors; however, putative disease‐modifying therapies (DMTs) like mABs are expected to alter the underlying rate of progression, potentially reducing our ability to detect effects of other DMTs on top of mABs. In addition, if the mechanism of action (MOA) also targets amyloid, the treatment effect may be further reduced by co‐administration (antagonism). Alternatively, treatments that target complementary mechanisms may actually perform better (synergy). This new context requires careful consideration when powering clinical trials.

**Method:**

We consider two clinical trial design scenarios. The first is a two‐arm trial to test a treatment as an add‐on to a mAB. The second is a four‐arm combination trial to represent the scenario where the mAB is started at the same time as investigational treatment. We calculate the required sample sizes for studies in three different patient populations: secondary prevention (MSDR = 0.34, 2‐year study), early AD (MSDR = 0.55, 18‐month study), and mild‐to‐moderate AD (MSDR = 0.67, 1‐year study). We consider scenarios with additivity, 30% antagonism, and 30% synergy.

**Result:**

As shown in the table, the expected interaction with mAB treatment can have a large effect on study design decisions, with antagonistic treatment effects requiring more than three times the sample size of a synergistic treatment in most scenarios. The four‐arm scenario also usually requires around a threefold increase in sample size compared to a simple add‐on study.

**Conclusion:**

Studies evaluating investigational therapies as add‐on to mABs are complex, and their cost to run will depend on how interactive the two treatments are. Treatments that are likely to work better with amyloid removed will be easier to study in this new context due to their complementary MOA. Symptomatic treatments may require fewer additional subjects than disease‐modifying treatments given the same expected effect size in the absence of mABs.